# Intra-arterial verapamil improves functional outcomes of thrombectomy in a preclinical model of extended hyperglycemic stroke

**DOI:** 10.3389/fphar.2023.1161999

**Published:** 2023-04-13

**Authors:** Sanaz Nasoohi, Parsa Alehossein, Masoumeh Jorjani, Candice M. Brown, Tauheed Ishrat

**Affiliations:** ^1^ Neuroscience Research Center, Shahid Beheshti University of Medical Sciences, Tehran, Iran; ^2^ Department of Neuroscience, School of Medicine, and Rockefeller Neuroscience Institute, West Virginia University, Morgantown, WV, United States; ^3^ School of Medicine, Tehran University of Medical Sciences, Tehran, Iran; ^4^ Department of Pharmacology, School of Medicine, Neurobiology Research Center, Shahid Beheshti University of Medical Sciences, Tehran, Iran; ^5^ Department of Anatomy and Neurobiology, School of Medicine, University of Tennessee Health Science Center, Memphis, TN, United States

**Keywords:** hyperglycemic stroke, verapamil, TXNIP, thrombectomy, large vessel occlusion

## Abstract

The abrupt hyperglycemic reperfusion following thrombectomy has been shown to harm the efficacy of the intervention in stroke patients with large vessel occlusion. Studies of ours and others have shown thioredoxin-interacting protein (TXNIP) is critically involved in hyperglycemic stroke injury. We recently found verapamil ameliorates cerebrovascular toxicity of tissue plasminogen activators in hyperglycemic stroke. The present study aims to answer if verapamil exerts direct neuroprotective effects and alleviates glucose toxicity following thrombectomy in a preclinical model of hyperglycemic stroke. Primary cortical neural (PCN) cultures were exposed to hyperglycemic reperfusion following oxygen-glucose deprivation (OGD), with or without verapamil treatment. In a mouse model of intraluminal stroke, animals were subjected to 4 h middle cerebral artery occlusion (MCAO) and intravenous glucose infusion. Glucose infusion lasted one more hour at reperfusion, along with intra-arterial (i.a.) verapamil infusion. Animals were subjected to sensorimotor function tests and histological analysis of microglial phenotype at 72 h post-stroke. According to our findings, glucose concentrations (2.5–20 mM) directly correlated with TXNIP expression in OGD-exposed PCN cultures. Verapamil (100 nM) effectively improved PCN cell neurite growth and reduced TXNIP expression as well as interaction with NOD-like receptor pyrin domain-containing-3 (NLRP3) inflammasome, as determined by immunoblotting and immunoprecipitation. In our mouse model of extended hyperglycemic MCAO, i.a. verapamil (0.5 mg/kg) could attenuate neurological deficits induced by hyperglycemic stroke. This was associated with reduced microglial pro-inflammatory transition. This finding encourages pertinent studies in hyperglycemic patients undergoing thrombectomy where the robust reperfusion may exacerbate glucose toxicity.

## Introduction

Hyperglycemia is a common manifestation in the acute phase of ischemic stroke. Observational studies have shown that hyperglycemia is associated with less favorable outcomes in stroke patients treated with either thrombolysis (Masrur et al., 2015; Cheng et al., 2020) or endovascular mechanical thrombectomy (MT) (Goyal et al., 2018; Kim et al., 2018). Mechanical thrombectomy is the preferential intervention to alleviate stroke-related death and disability (Smith et al., 2009; Malhotra et al., 2017) in stroke patients with large vessel occlusions (Kang et al., 2013). Furthermore, MT provides extended (≥6-h) time windows compared to thrombolysis and is in the spotlight in stroke patients (Hendrix et al., 2021). However, emerging evidence indicates the immediate recanalization following the MT procedure amplifies the detrimental influence of hyperglycemia and deters MT outcomes in stroke patients (Chamorro et al., 2019). Based on the existing knowledge, restoration of blood flow with high glucose concentration promotes tissue acidosis and the production of reactive oxygen and nitrogen species that increase infarct size, brain swelling, and blood–brain barrier disruption (Hafez et al., 2015). Recent findings by us and others have indicated that high intracellular glucose concentration induces thioredoxin-interacting protein (TXNIP) (Kim et al., 2012). TXNIP mediates hyperglycemia-induced oxidative stress (Schulze et al., 2004) while stimulating the NOD-like receptor protein (NLRP3) inflammasome assembly, leading to inflammatory responses in the immune cells (Nasoohi et al., 2018; Ismael et al., 2021a).

Verapamil, an L-type calcium channel blocker, has been recently shown to repress TXNIP expression (Zhou et al., 2018; Xu et al., 2019). According to our earlier findings, intravenous (i.v.) verapamil attenuates tPA-induced BBB injury and TXNIP/NLRP3 activation in a mouse model of hyperglycemic stroke (Ismael et al., 2021b). It is uncertain though, if verapamil may efficiently ameliorate the toxic effect of robust glucose overflow to the ischemic region following MT. Intra-arterial (i.a.) verapamil is a safe and routine practice to treat cerebral vasospasm, and is less likely to lower perfusion pressure (Forshay et al., 2021) which is detrimental for the ischemic tissue in a stroke brain. The study by Bix and others (2017) has recently shown that the selective i.a. administration of verapamil permits for a wide therapeutic index in ischemic stroke, modeled in normoglycemic mice for 65 min (Fraser et al., 2017).

According to the most recent updates, the Stroke Treatment Academic Industry Roundtable (STAIR) has recommended preclinical studies to consider the maximum delay to treatment after stroke (Lyden et al., 2021). Although a preclinical model of thrombectomy is not yet implemented in small rodents (Gralla et al., 2006), filament withdrawal following the standard intraluminal stroke is applicable to simulate mechanical clot removal following embolic stroke (Messmer et al., 2022). Therefore, we implemented an extended mouse model of hyperglycemic filament ischemic stroke to simulate the majority of stroke patients receiving recanalization therapy after a clinically relevant long period of arterial occlusion (Messmer et al., 2022). In our initial *in vitro* experiments, we found severe hyperglycemia may hinder verapamil protection against oxygen-glucose deprivation (OGD) in primary neural cells. To achieve ideal efficacy, we maintained an i.a. verapamil infusion for the first hour of hyperglycemic reperfusion following the extended hyperglycemic ischemic stroke.

## Materials and methods

Regarding the lack of enough information on how verapamil directs efficacy in hyperglycemic conditions, the protective effects were initially tested in mouse primary neural cultures subjected to hyperglycemic OGD. With the abrupt re-oxygenation and media replacement, compared to chemical (i.e., cyanide and cobalt) models of hypoxic injury *in vitro*, OGD is particularly suitable to study neural injury in response to immediate reperfusion at mechanical recanalization. To address the main hypothesis in a clinically relevant model, intra-arterial verapamil was then examined in filament stroke in mice with close similarities to stroke patients.

### Primary cultures of cortical neurons

As previously described (Cao et al., 2003), cultures of primary cortical neurons (PCNs) were prepared from E17 mouse brains. In brief, the brains were isolated, cortices were dissected, and the cells were dissociated in HBSS (Hank’s Buffer Salt Solution) following 20 min trypsinization at 37°C. The cell suspensions were passed through a cell strainer (0.4 µM pore). The filtrate was then cleaned from cellular debris by passing through a BSA cushion (3%) followed by centrifuging at 400 rpm. The pellets were suspended in DMEM-F12 supplemented with 10% FBS and seeded in Poly-L-Lysine (4707, Sigma) coated plates in a density of 100,000 cells per cm^2^ in Neurobasal medium (NBM; Invitrogen, Thermo Fisher Scientific) supplemented with 25 mM glutamate, 0.5 mM L-glutamine, and 2% B27 supplement (Invitrogen, Thermo Fisher Scientific), in 6- or 24-well plates. After 3 days incubation at 37°C in humidified 95% air and 5% CO_2_, the medium was partially changed every other day till day 14 post-dissection, when cultures consisted primarily of mature neurons.

## Oxygen-glucose deprivation and treatments

After 14 days, *in vitro* (DIV) PCN cultures were exposed to oxygen-glucose deprivation (OGD). The culture medium was replaced with balanced salt solution (BSS, in mmol/L: 116 mM NaCl, 5.4 mM KCl, 0.8 mM MgSO_4_, 1 mM NaH_2_PO_4_·2H_2_O, 262 mM NaHCO_3_, 1.8 mM CaCl_2_, pH 7.2, <0.1% O_2_), previously saturated with 95% N_2_/5% CO_2_ at 37°C. Cultures were then placed in a hypoxic incubator chamber (STEMCELL Inc., United States), which was flushed for 5 min with 95% N_2_/5% CO_2_ and placed in a water-jacketed incubator at 37°C for 90 min. PCN cultures were then returned to 95% air, 5% CO_2_, with serum-free medium (glucose-free RPMI, penicillin/streptomycin 1%) containing different glucose concentrations (2.5, 5, 20, or 37.5 mM) for 24 h. PCN cultures were exposed to hyperglycemic reperfusion (glucose 20 or 37.5 mM) and were treated with verapamil (in 0, 10, 100, or 1000 nM concentration).

### Neural viability

PCN 24-well culture plates were incubated for 24 h at 37°C under different verapamil dosages. Cell viability was measured by the 3-(4,5-dimethylthiazol-2-yl)-2,5-diphenyltetrazolium bromide (MTT) (Biomol Inc., United States) reduction method. MTT was added to the culture media to a final concentration of 0.5 mg/mL and incubated for 3 h under standard conditions. The purple formazan product was then solubilized using DMSO, and the absorbance was measured using a microplate reader. Neurite length was also analyzed, as described earlier (Nasoohi et al., 2023). Neurons were fixed with ice-cold 4% PFA, incubated with rabbit anti-MAP2 (1:150, Abcam) goat anti-rabbit IgG antibody, CY^®^3 (Vector Laboratories, CA, United States) in TBS containing Triton X (0.1% Triton X-100) and BSA (1%). Cells were then washed and mounted with Vectashield HardSet (Vector Laboratories, CA, United States). Images were captured using an inverted fluorescent microscope (Olympus) and analyzed using ImageJ software (NeuronJ plugin) (*N* = 6 per group).

### Western blot

PCNs were scraped from plates 24 h following OGD in ice-cold NP-40 lysis buffer (150 mM sodium chloride, 50 mM Tris pH 8.0, 1.0% NP-40). Standardized samples (25 μg protein) were equally loaded for SDS gel electrophoresis and transferred to the PVDF membrane (Merck, IPVH00010). Membrane proteins were then probed with mouse anti-TXNIP antibody (1/2000, Novous biological) or rabbit anti-β-actin (1/3000, Cell Signaling) overnight (4°C) and incubated with HRP-linked anti-mice IgG (1/10,000, Sigma) and anti-rabbit IgG (1/3000, Cell Signaling) antibodies. Specific protein bands were visualized using the ECL Select detection reagent (Amersham, United States), and the acquired images were analyzed with ImageJ software.

### Immunoprecipitation

PCNs were lysed in NP40 buffer through mild sonication, and equal amounts of proteins (100 μg) were incubated with 3 μg of anti-NLRP3 antibody (Novus Biologicals, United States) at 4°C overnight. Bound proteins were then recovered after the addition of 15 μL of pre-washed anti-rabbit IgG-magnet beads (Rockland, United States) for 1 h at 4°C. Beads were then gently washed five times, followed by elution of bound proteins from the beads in a Laemmli buffer. Bound proteins were then analyzed by Western blot with polyclonal anti-TXNIP or NRLP3 antibodies (both obtained from Novus Biologicals).

### Surgical procedure and treatments

Animal care and use were performed in accordance with the National Institute of Health (NIH) guidelines for research in animals and approved by the Institutional Animal Care and Use Ethics Committee (IR.SBMU.PHNS.REC.1399.146). Adult male mice (4 weeks old, 25 ± 1 g) were anesthetized with isoflurane (2%–5%) in a mixture of 70% N_2_O/30% O_2_ using an inhalational anesthesia machine (Parkland Scientific, Coral Springs, FL, United States). Under a surgical microscope, the left jugular vein was cannulated by a modified polyethylene tubing (PE10, Braintree Sci, United States). Ischemic stroke was then induced by the standard right middle cerebral artery occlusion (MCAO) as previously described (Simani et al., 2017). Slight modifications were implemented to our mouse model with subsequent insertion of the stroke filament and verapamil i.a. microcatheter into the ICC lumen. A tiny puncture was made in the proximal trunk of the right internal common carotid (ICA) through which a silicon-coated suture (Doccol, Sharon, MA, United States) was inserted and advanced to the origin of MCAO. The filament was secured in the artery by a 6–0 silk suture. Immediately after occlusion, glucose i.v. infusion was started through a syringe connected to a micro-infusion pump (25% w/v, 40 µL/h), and animals were allowed to recover from anesthesia. Animals with blood glucose below 15 mM were excluded from further studies. Ten minutes before the scheduled time for the filament withdrawal (4 h) ends (∼220 min post occlusion), the animals were again anesthetized. A small hole was made in the common carotid artery at least 5 mm before bifurcation, and the tip of the i.a. verapamil microcatheter (PI-FL-191, Doccol, United States) was inserted, advanced to the bifurcation before the stroke filament puncture, and loosely fixed using a 6–0 silk suture. The filament was then gently withdrawn from ICA (at 4h post-occlusion). Immediately after, the verapamil microcatheter was advanced to ICA, and verapamil micro-infusion (0.5 mg/kg, 40 µL/h) was started. Care was taken so that the verapamil microcatheter did not reach the MCAO origin and impair the perfusion. After one hour under anesthesia, both glucose and verapamil microcatheter were gently withdrawn. Intensive inter-/post-operation care was taken to support enough hydration, as recently described (Messmer et al., 2022).

### Neurological deficit tests

A battery of motor function scoring was used to assess the benefit of i.a. verapamil in animals that underwent extended hyperglycemic stroke: Bederson’s score, the forepaw grasp test (Ishrat et al., 2019), and the modified neurological severity score (mNSS). All behavioral assessments were performed with an experimenter blinded to the experimental groups. A modified forepaw test was used to assess the animal’s ability to grasp a horizontal bar when held by the base of the tail. The mice were assigned a score of 0 if both of the forepaws were able to grasp the bar immediately; a score of 0.5 if the right forepaw grasped the bar with decreased grip strength; a score of 1 if the right forepaw grasped the bar with delayed motion and decreased strength; a score of 1.5 if the right forepaw was not able to grasp the bar; and a score of 2 if the rat displayed no motion/attempt to grasp the bar. A Bederson’s scoring system was also used to indicate if there is no neurological deficit (1), or if the animal shows flexion of the contralateral forelimb (1); severe forelimb flexion and decreased resistance to lateral push without circling (2); unidirectional circling (3); loss of spontaneous motor activity (4); and death due to stroke (5). We also compared animals’ motor and reflex functions based on a 16‐score scale mNSS adopted from earlier studies with sight modification (Supplementary Table S1) (Lee et al., 2013).

### Microglial morphology analysis

In brief, the cryo-protected brain samples were sectioned into 20-micron free-floating sections, permeabilized with 20% methanol, blocked with 10% normal goat serum (NGS, Thermo fisher), and incubated with anti-Iba-1 primary antibody (1/200, Abcam, United States) overnight at 4°C. The samples were then incubated with HRP‐conjugated secondary antibody for 1 h and were tested for 3,3′‐diaminobenzidine tetrahydrochloride (DAB) immunoreactivity using the substrate kit (DS-SK-4100, Vector Laboratories, United States). After the sections were mounted on slides, replicate images were acquired for each experimental group (*n* = 4) using a bright field microscope.

### Statistical analysis

The results were expressed as mean ± SEM. Differences among experimental groups were evaluated by Student’s two-tailed *t*-test or ANOVA followed by Tukey’s *post hoc* test. Significance was defined by *p* < 0.05.

## Results

### Glucose promotes OGD-induced TXNIP expression in primary neurons

**FIGURE 1:**
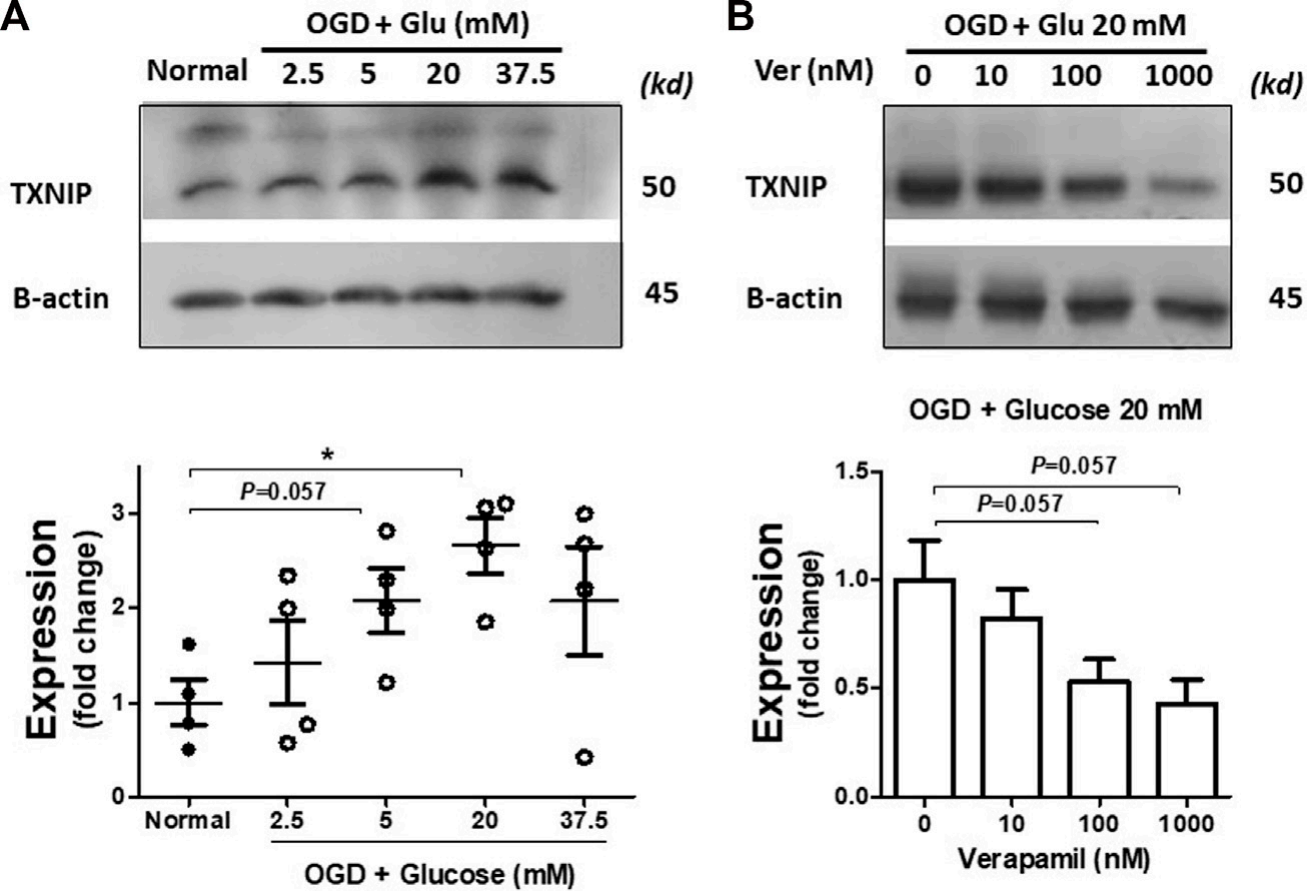
Verapamil decreases TXNIP upregulation induced by OGD-HGr. PCN cultures were exposed to OGD and incubated in normal conditions with increasing glucose concentrations (2.5, 5, 20, and 37.5 mM) for 24 h. Normal and OGD-HGr PCN cultures were then immunoblotted for TXNIP analysis. TXNIP was upregulated following OGD in correlation with glucose levels (2.5, 5, and 20 mM) **(A)**. Verapamil addition to the medium (0, 10, 100, or 1000 nM) could reduce TXNIP expression in OGD-HGr PCN cultures **(B)**. TXNIP, thioredoxin-interacting protein; PCN, primary neural cells; HGr, hyperglycemic reperfusion; OGD, oxygen-glucose deprivation. All values are presented as mean ± SEM (*n* = 4/group); *p < 0.05.

PCNs were exposed to incremental glucose concentrations following OGD. According to immunoblotting data, OGD induced discernible TXNIP upregulation in normal glucose levels as simulated with 5 mM glucose concentration in the medium (Figure 1A), which is consistent with previous reports indicating ischemic injury enhances TXNIP expression (Kim et al., 2012). TXNIP upregulation was gradually amplified with increasing glucose concentration up to 20 mM (*p < 0.05*), reflecting clinically relevant severe hyperglycemia (Lindsberg and Roine, 2004). However, in 37.5 mM, the typical hyperglycemic condition in accelerated *in vitro* studies (Wang et al., 2019), we could not detect uniform TXNIP upregulation. Based on this primary information, 20 mM glucose (around 370 mg/dL) was selected as the specific concentration for further hyperglycemic *in vitro* experiments.

### Enhanced neural TXNIP expression and interaction with NLRP3 subside with verapamil

**FIGURE 2:**
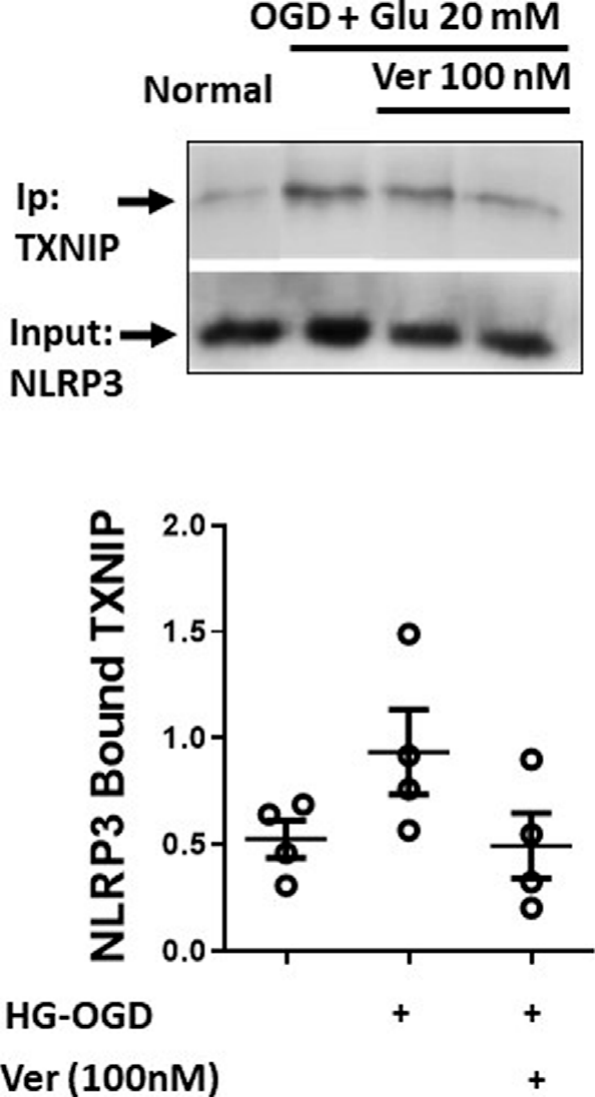
Verapamil marginally reduces TXNIP binding to NLRP3 inflammasome in OGD-HGr. PCN cultures were exposed to OGD and incubated in normal conditions with glucose concentration of 20 mM in medium for 24 h. OGD-HGr PCN cultures were treated with verapamil in 0 and 100 nM concentration. TXNIP in PCN cells extracts were immunoprecipitated with NLRP3-specific antibody using magnet beads. The samples were then immunoblotted for the precipitated TXNIP and the NLRP3 as the input molecule. TXNIP, thioredoxin-interacting protein; NLRP3, NOD-like receptor protein 3; PCN, primary neural cells; HGr, hyperglycemic reperfusion; OGD, oxygen-glucose deprivation; ip., immunoprecipitated. All values are presented as mean ± SEM (*n* = 4/group); *p < 0.05.

PCNs exposed to hyperglycemic reperfusion following OGD (OGD-HGr) were treated with exponentially increasing concentrations of verapamil in the medium (Figure 1B). According to immunoblots and densitometry analysis (n = 4 per group), we could determine a tangible suppression in TXNIP induced by OGD-HGr in both 100 and 1000 nM verapamil concentrations, close to statistically significant levels (*p = 0.057*). TXNIP was further studied for its capacity to bind to NLRP3 inflammasome in hyperglycemic conditions. According to obtained data (Figure 2), NLRP3 inflammasomes bound to more TXNIP molecules in OGD-HGr compared to that in normal PCNs, when equal NLRP3 molecules (input) were co-immunoprecipitated with TXNIP (ip.). Verapamil (100 nM) exposure could marginally reduce the interaction between TXNIP and NLRP3 in neural cells subjected to OGD-HGr.

### Verapamil provides direct neuroprotection in OGD with hyperglycemic reperfusion

**FIGURE 3:**
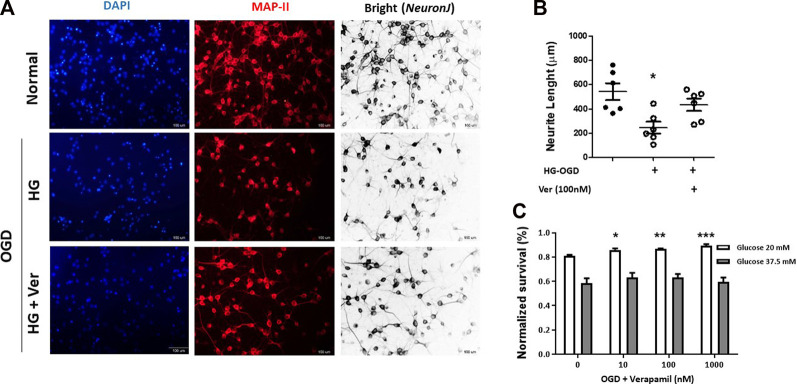
Verapamil improved cell survival and neurite growth in OGD-HGr PCN cultures. PCN cultures were exposed to OGD and incubated in normal conditions with glucose concentration of 20 mM in medium for 24 h with or without verapamil. To analyze neurite growth, PCN cells were probed for MAP-II antigen, and average neurite lengths were measured in 1 mm^2^ fields. Verapamil (100 nM) could partially improve neurite length in OGD-HGr PCN cells **(A)**. To address OGD-HGr PCN cell viability, MTT assay was performed in cultures treated with verapamil (0, 10, 100, or 1000 nM) for 24 h, either in 20 or 37.5 mM glucose concentrations **(B)**. PCN, primary neural cells; HGr, hyperglycemic reperfusion; OGD, oxygen-glucose deprivation. All values are presented as mean ± SEM (*n* = 6–10/group). *p < 0.05, **p < 0.01, ***p < 0.001 compared to control.

Direct neuroprotective effects of verapamil were analyzed in hyperglycemic reperfusion injury to PCNs (Figure 3). Although verapamil did not change PCN numbers, it induced a significant increase in average neurite length (Figure 3A, B). In earlier studies, verapamil treatment has been shown to preserve neurite injury in micromolar concentrations (Maniskas et al., 2016). Based on MTT assay data, verapamil could also produce significant dose-dependent protection in OGD PCNs exposed to 20 mM rather than 37.5 mM concentration, where severe hyperglycemia and oxidative stress remarkably compromise normal mitochondrial reduction capacity (Figure 3C). However, verapamil-induced neuroprotection persisted with 20 mM glucose treatment, and gradually declined at verapamil concentrations above 1000 nM (data not shown).

### Intra-arterial verapamil attenuates neurological deficit in extended hyperglycemic stroke

**FIGURE 4:**
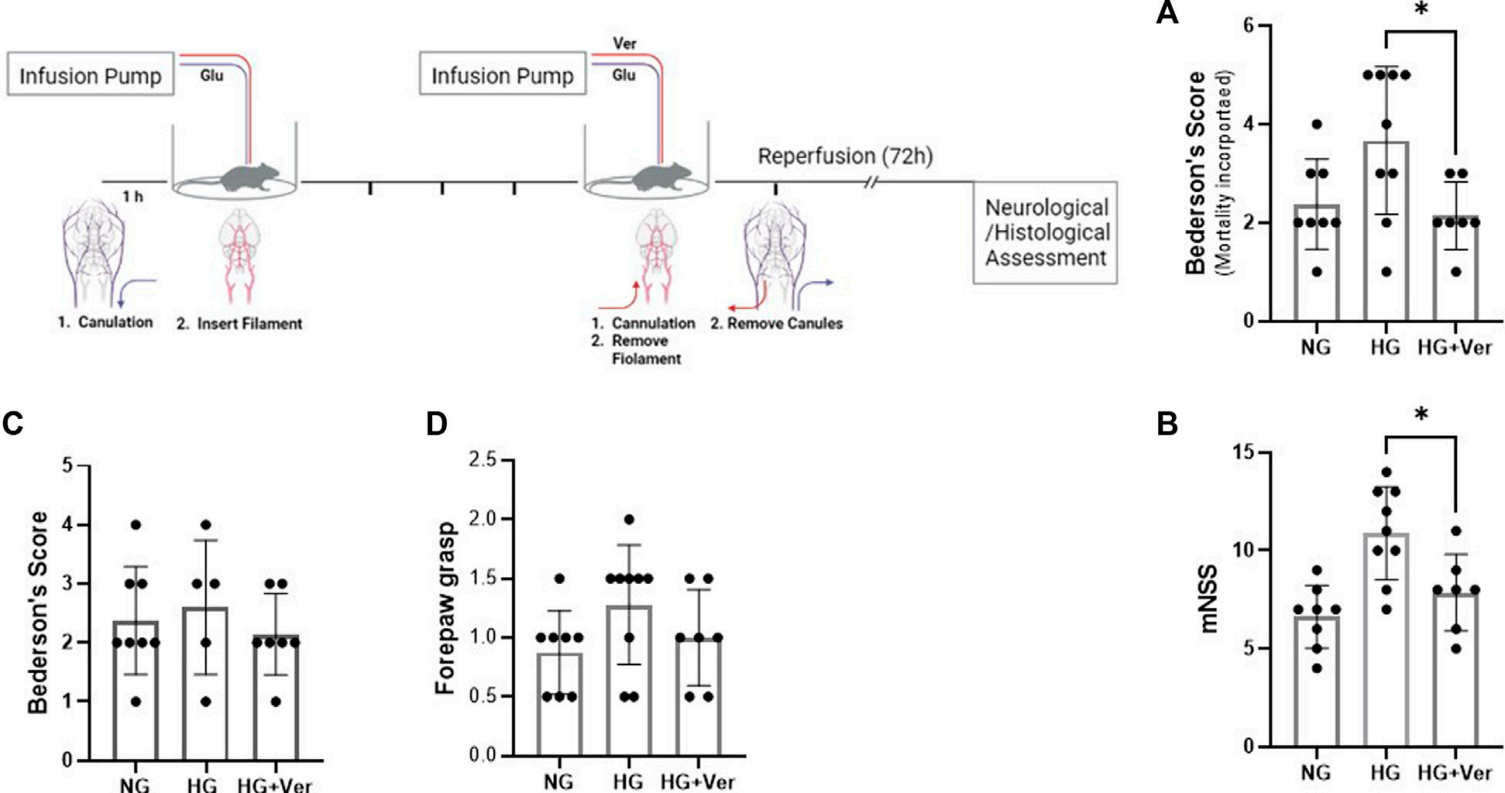
Intra-arterial verapamil attenuates neurological deficits in a preclinical model of thrombectomy in hyperglycemic stroke. Adult male mice were subjected to 4 h intraluminal MCAO. Hyperglycemia was established with intravenous glucose infusion starting from occlusion time till 1 h post-filament withdrawal. Glucose infusion (25%, 40 µL/h) lasted one more hour after blood flow restoration, along with intra-arterial (i.a.) verapamil infusion. Hyperglycemic stroke mice represented lower Bederson’s scores **(A)** and mNSS **(B)** when treated with i.a. verapamil (0.5 mg/kg, 40 µL/h, 1 h). The animals also showed a trend toward lower Bederson’s score after excluding stroke-induced deaths **(C)**. The scores obtained in forepaw grasp also showed a tendency toward milder functional deficits **(D)**. MCAO, middle cerebral artery occlusion; Ver, verapamil; Glu, glucose; HG, hyperglycemic; NG, normoglycemic; mNSS, modified neurological severity score. All values are presented as mean ± SEM (*n* = 7–9); *p < 0.05.

To establish hyperglycemic stroke injury, we adopted the initial glucose dosage from earlier studies (Wei et al., 2003) and performed a dose-response experiment to scale down the mortality rate (Table 1). Although animals showed significant weight loss following surgery (15.875 g ± 0.64 at 72h I/R), there was no discernible difference among NG, HG, and HG + Ver stroke mouse weight loss. Verapamil dosage was set according to our pilot studies. We started at 10 mg/kg dosage according to the reports on single i.a. verapamil dosage (Fraser et al., 2017) and then adjusted it to lower dosages that do not cause mortality in our i.a. verapamil-infused animals. According to the neurological assessments, i.a. verapamil could attenuate glucose-induced exacerbation in Bederson’s score (Figure 4A) as well as mNSS (Figure 4B). However, after excluding stroke-related deaths, the differences in Bederson’s score were not statistically significant but still showed a discernible trend toward the improved deficit score in verapamil animals (Figure 4C). According to our findings, the differences in animals’ mNSS were mostly reflected in the beam walk test (data not shown). Although we implemented a graded fore-paw grasp test, the scores did not show a significant difference between our experimental groups, probably because of the lack of enough sensitivity to detect the differences (Figure 4D).

**TABLE 1:** Blood glucose and mortality rate in response to i.v. glucose infusion in 4 h MCAO mice.

Infusion rate (pL/h)	Blood glucose (mg/dL)	Mortality
100	339.65 ± 149.57	7 of 7
60	282.52 ± 94.55	4 of 6
40	201.88 ± 46.94	2 of 9

### Neuroprotective effects of verapamil associates with reduced microglial activation

**FIGURE 5:**
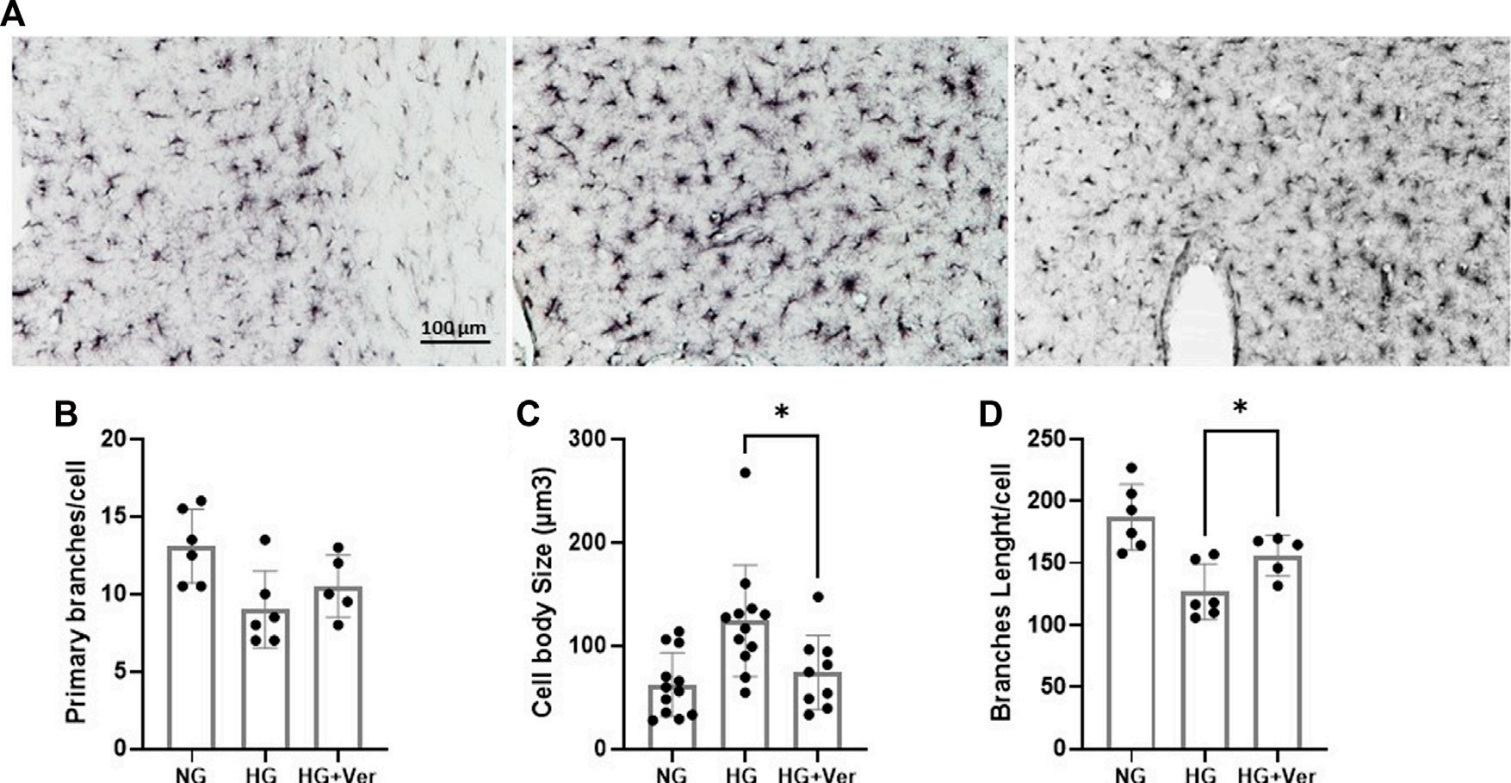
Intra-arterial verapamil mitigates microglial inflammatory transition following thrombectomy in a preclinical model of hyperglycemic stroke. Adult mice were subjected to 4 h intraluminal MCAO. Hyperglycemia was established with intravenous glucose infusion (25%, 40 µL/h) starting from occlusion time till 1 h post-filament withdrawal. Glucose infusion was continued for one more hour at reperfusion, along with intra-arterial (i.a.) verapamil infusion (0.5 mg/kg, 40 µL/h, 1 h). As demonstrated in the representative images **(A)**, The number of primary branches following stroke did not change with prolonged hyperglycemia (5 h) or i.a. verapamil infusion (1 h) **(B)**. Prolonged hyperglycemia encouraged pro-inflammatory phenotype in penumbral microglia, as indicated by microglial hypertrophy **(C)** and reduced total branches length/cell **(D)**. The corresponding transitions to inflammatory phenotype were partially prevented by i.a. verapamil. MCAO, middle cerebral artery occlusion; Ver, verapamil; Glu, glucose; HG, hyperglycemic; NG, normoglycemic. All values are presented as mean ± SEM (*n* = 3–4); *p < 0.05.

Microglial response to glucose toxicity is an elaborate and context-sensitive process. Earlier studies have shown that streptozocine (STZ)-induced high glucose levels increase the number of active microglia in the hippocampus (Wanrooy et al., 2018). High blood glucose in mouse models of type 2 diabetes has also been shown to induce a remarkable microglial transition to a pro-inflammatory phenotype (Tsubaki et al., 2020). On the other hand, a recent report concludes that STZ-induced hyperglycemia (>16.7 mM) inhibited microglia activation following 30 min of MCAO (Guo et al., 2021). However, this may be a consequence of reduced glucose influx for insufficient insulin signaling. Interestingly in this line, our non-diabetic mice, who could not establish a high blood glucose level upon infusion, showed high mortality, even before MCAO surgery (data not shown). According to our immunostaining analysis (Figure 5A), the alterations in the morphology of cortical microglia in our hyperglycemic non-diabetic stroke mice, however did not changes the branches number (Figure 5B); caused a strong shift toward pro-inflammatory phenotype, as indicated by the increase in hypertrophic cells (Figure 5C) and shorter branches (Figure 5D). The number of primary branches did not demonstrate a remarkable difference. The observed microglial activation, either as a direct effect of the intracellular glucose buildup or the consequence of aggravated I/R-induced BBB breakdown, was significantly mitigated by i.a. verapamil.

## Discussion

Hyperglycemia, with or without pre-existing diabetes, is linked to increased mortality and morbidity in stroke patients (Capes et al., 2001). Although insulin therapy is sought as a promising therapeutic approach (O’Connell et al., 2006), intensive glucose normalization has not shown enough efficacy in clinical trials (Bellolio et al., 2012; Johnston et al., 2019). With the growing interest in endovascular thrombectomy, there is an unmet need to implement measures to reduce the deteriorating effect of robust glucose toxicity in hyperglycemic subjects. According to our preclinical examinations, the calcium channel blocker verapamil is effective enough to improve the functional outcomes of thrombectomy in a translational model of hyperglycemic stroke. Our *in vitro* and *in vivo* examinations suggest i.a. verapamil modulates TXNIP/NRP3 inflammasome and controls the microglial transition to a pro-inflammatory phenotype.

Most of the existing knowledge on glucose toxicity emerges from studies on diabetic animals. Given the fact that glucose influx is impaired in diabetes at least partially, the findings may not be extrapolated to hyperglycemic non-diabetic subjects. Fewer studies have focused on glucose toxicity in non-diabetic stroke and searched for potential therapies like deferoxamine (Xing et al., 2009), pregabalin (Song et al., 2017), and 2-Deoxy-D-glucose (Wei et al., 2003). According to our earlier studies, tissue plasminogen activator (tPA), the admitted therapy in stroke patients, promotes TXNIP-NLRP3 inflammasome activation in hyperglycemic stroke mouse model (Ismael et al., 2020). Recently, we showed that verapamil intravenous administration significantly reduces tPA-induced injury and hemorrhagic transformation (Ismael et al., 2021b). To weigh the drug benefit against the abrupt glucose toxicity in a preclinical model of MT, we used an extended (4 h) intraluminal filament of stroke in mice that were awake with persisting hyperglycemia for 5 h.

Our designed model of hyperglycemic stroke attempts to mimic clinical conditions through a few major modifications in addition to the extension of the occlusion time. The earlier studies by us and others (Hafez et al., 2015; Hafez et al., 2016) have used a bolus glucose injection of glucose before MCAO to generate an accelerated model glucose toxicity with a 1-h to 2-h occlusion period. Our model demonstrates a steady i.v. glucose buildup after stroke to closely mimic stress hyperglycemia. Furthermore, we maintained animals in an awake state throughout most of the procedure to simulate stroke patients as well as eliminate the isoflurane effect on glucose metabolism and uptake (Constantinides et al., 2010). The intracerebral i.a. verapamil administration is also approved in clinical practice. According to our neurological assessments, the graded fore-paw grasp test was not sensitive enough to show any significant difference between the experimental groups. Our further functional assessments demonstrated that i.a. verapamil could attenuate glucose-induced exacerbation in either Bederson’s score or mNSS. The benefit of i.a. verapamil administration was more significant in reducing animals’ mortality and beam imbalance. This suggests that i.a. verapamil at reperfusion may ameliorate neurological deficits in the acute phase. However, our findings may not conclude the main underlying mechanism; verapamil may probably act through mitigating the microglial inflammatory transformation caused by glucose toxicity.

Intracellular high glucose concentrations induce TXNIP (Kim et al., 2012), which is known to mediate hyperglycemia-induced oxidative stress (Schulze et al., 2004; Bedarida et al., 2018; Ren et al., 2018) and to deter glucose availability to the neural cells in the brain (Waldhart et al., 2017). The incremental increase in glucose in our PCN culture medium enhanced the neural TXNIP level following OGD and moderately reduced TXNIP/NLRP3 binding. These effects did not sustain in intensive hyperglycemia, where the function of biomolecules may not preserve the signaling. Importantly, our dose-response experiments showed that in the same high glucose levels, verapamil may not preserve enough efficacy to ameliorate glucose toxicity in OGD. This finding may support the hypothesis that functional TXNIP-NRP3 signaling is required for verapamil-mediated direct neuroprotection against hyperglycemic ischemic reperfusion (I/R) injury.

According to our *in vitro* findings, verapamil could effectively reduce the toxic effect of moderate hyperglycemia in OGD-exposed primary neurons. This effect paralleled a significant reduction in TXNIP expression and a moderate decrease in TXNIP/NLRP3 interaction. These findings, however, may not conclude that neural TXNIP mediates verapamil benefits in our hyperglycemic stroke mice. In this connection, a recent *in vitro* study suggests that microglial NADPH oxidase, but not neural calcium channels, mediates the neuroprotective effects of verapamil (Liu et al., 2011). Experimental evidence indicates verapamil control over microglial phenotype is attributed to the blockage of L-type voltage-dependent Ca^2+^ channels. Intracellular Ca^2+^ regulates microglia transition to an activated immune-effector state by interacting with downstream effectors (e.g., calmodulin and calcineurin) to promote pro-inflammatory cytokines and chemotaxis (Hopp, 2021). Verapamil-induced decrease in intracellular Ca^2+^ has also been recently shown to repress TXNIP transcription and subsequent NLRP3 inflammasome activity (Zhou et al., 2018; Xu et al., 2019) through carbohydrate response element-binding protein (Xu et al., 2012). Consistently, our morphological studies showed verapamil could significantly prevent microglial activation by hyperglycemic I/R injury; that is, stroke brains showed enhanced microglial swelling in response to glucose toxicity which was moderately controlled in those that received i.a. verapamil. This was associated with the elongation of microglial branches and a subtle rise in the branching number, which is the characteristic of less activated more ramified microglia. However, our immunostaining images did not permit us to analyze and compare the number of microglial final processes endpoints to determine the activated phenotype (Abdul et al., 2021). Our findings may not conclude whether verapamil preserves microglia phenotype through repression of TXNIP, a well-defined effector for high glucose to drive inflammasome assembly and microglial inflammatory behavior (Tsubaki et al., 2020).

## Data Availability

The raw data supporting the conclusion of this article will be made available by the authors, without undue reservation.
