# Supplementary material for: Intra-arterial verapamil improves functional outcomes of thrombectomy in a preclinical model of extended hyperglycemic stroke
Source: Front Pharmacol. 2023 Apr 13;14:1161999. doi: 10.3389/fphar.2023.1161999 (PMC10134451; doi:10.3389/fphar.2023.1161999)
Supplement: Supplementary file 1 [file Image1.PDF]

Supplementary Material

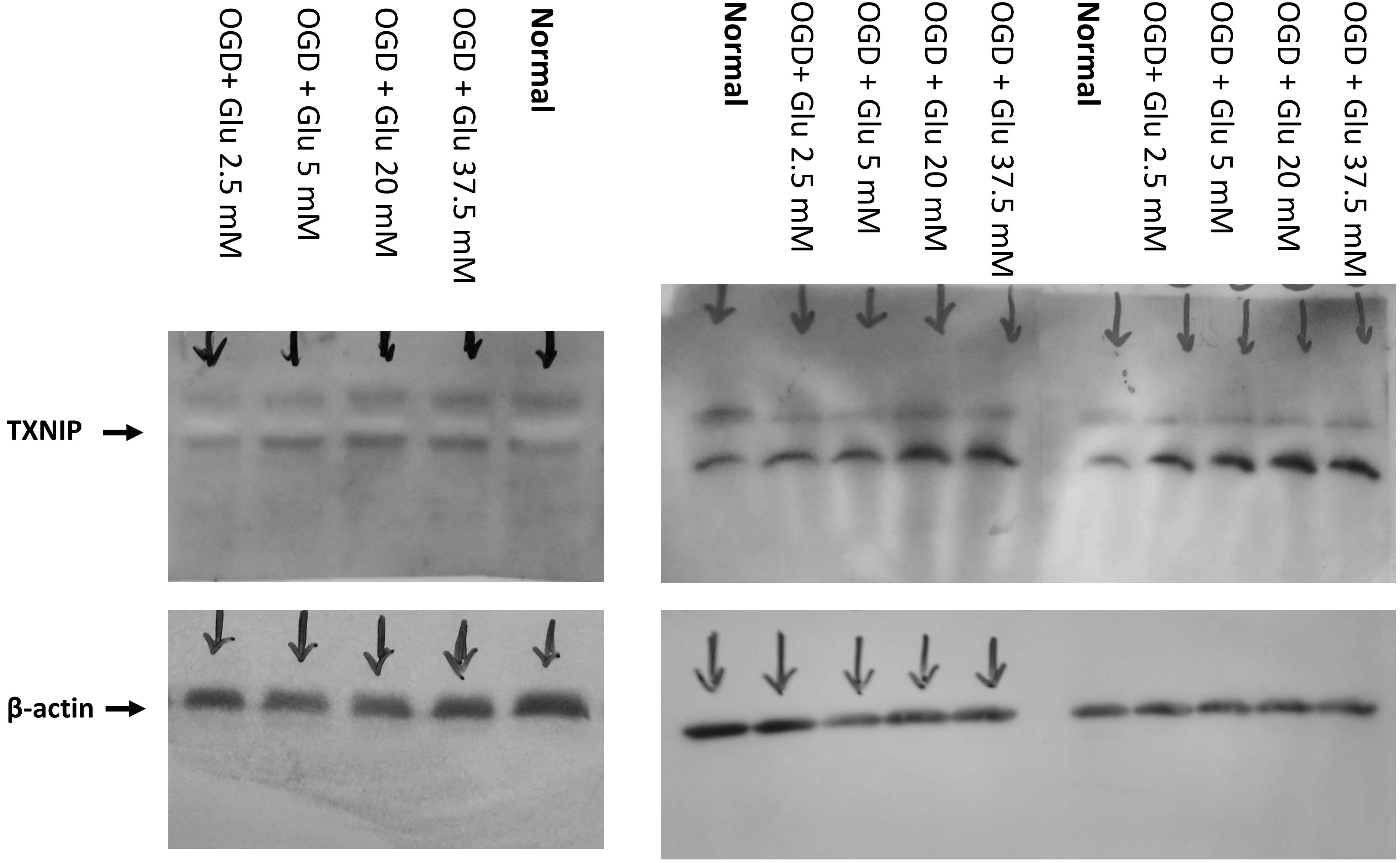

Fig 1A immunoblots:
TXNIP expression

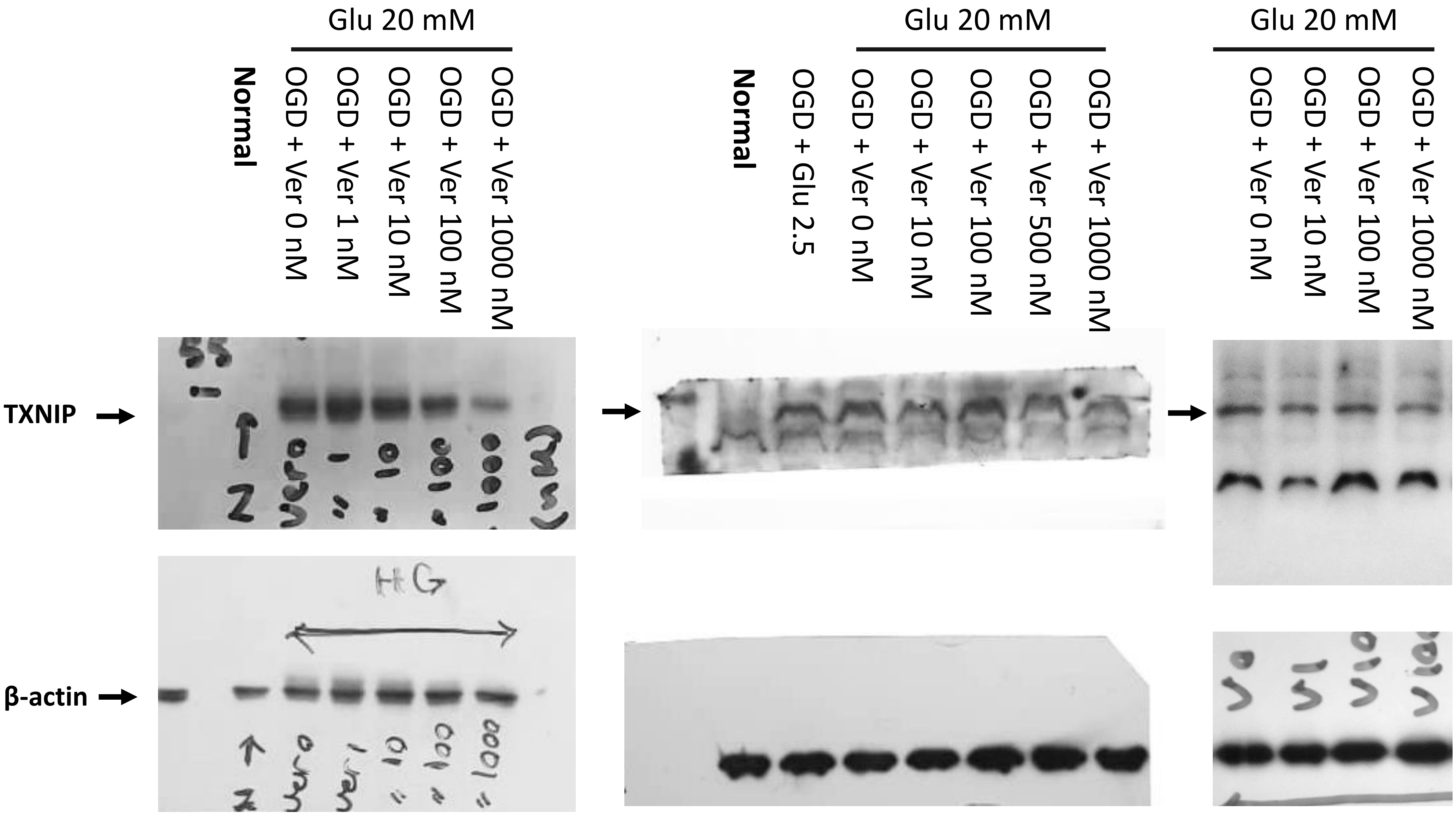

Fig 1B immunoblots:
TXNIP expression

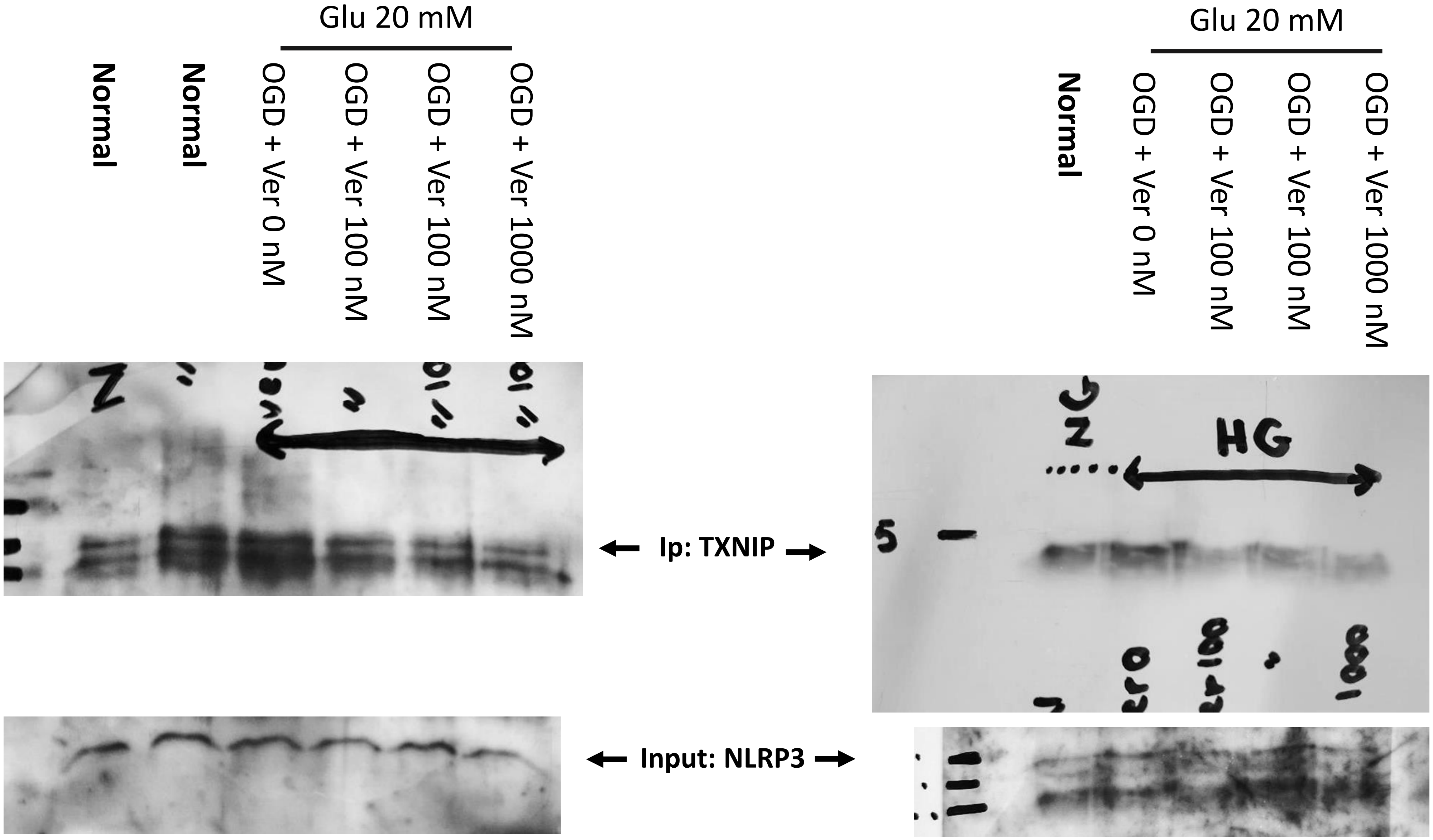

Fig 3 immunoblots (A):
TXNIP/NLRP3 interaction

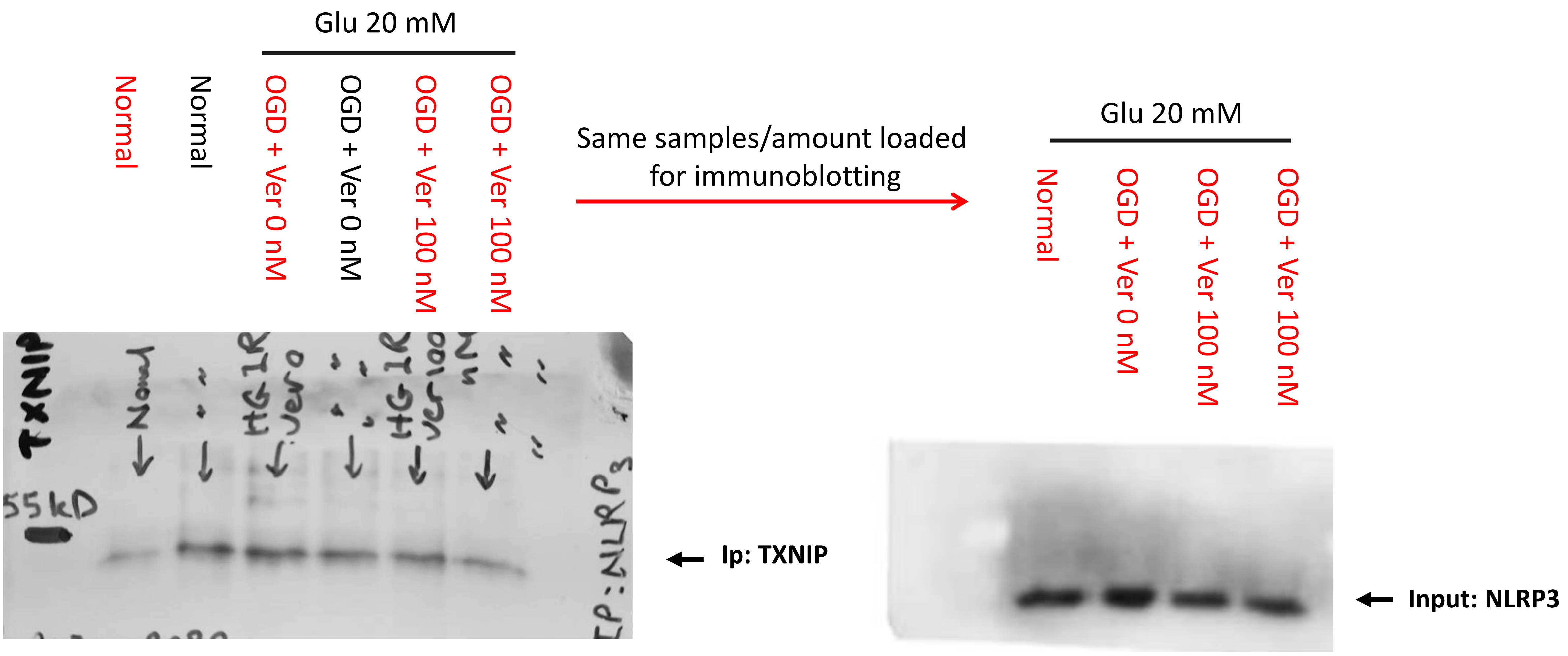

Fig 3 immunoblots (B):
TXNIP/NLRP3 interaction
